# Long-Term Outcomes and the Post-Intensive Care Syndrome in Critically Ill Children: A North American Perspective

**DOI:** 10.3390/children8040254

**Published:** 2021-03-24

**Authors:** Alan G. Woodruff, Karen Choong

**Affiliations:** 1 Department of Anesthesiology, Section of Pediatric Critical Care Medicine, Wake Forest University School of Medicine, Winston-Salem, NC 27157, USA; 2 Department of Pediatrics, Division of Critical Care, McMaster University, Hamilton, ON L8S 4K1, Canada; choongk@mcmaster.ca; 3 Department of Health Research Methods, Evidence and Impact, McMaster University, Hamilton, ON L8S 4K1, Canada; choongk@mcmaster.ca

**Keywords:** pediatric intensive care, pediatric critical illness, post-intensive care syndrome, outcomes, long-term outcomes

## Abstract

Advances in medical and surgical care for children in the pediatric intensive care unit (PICU) have led to vast reductions in mortality, but survivors often leave with newly acquired or worsened morbidity. Emerging evidence reveals that survivors of pediatric critical illness may experience a constellation of physical, emotional, cognitive, and social impairments, collectively known as the “post-intensive care syndrome in pediatrics” (PICs-P). The spectrum of PICs-P manifestations within each domain are heterogeneous. This is attributed to the wide age and developmental diversity of children admitted to PICUs and the high prevalence of chronic complex conditions. PICs-P recovery follows variable trajectories based on numerous patient, family, and environmental factors. Those who improve tend to do so within less than a year of discharge. A small proportion, however, may actually worsen over time. There are many gaps in our current understanding of PICs-P. A unified approach to screening, preventing, and treating PICs-P-related morbidity has been hindered by disparate research methodology. Initiatives are underway to harmonize clinical and research priorities, validate new and existing epidemiologic and patient-specific tools for the prediction or monitoring of outcomes, and define research priorities for investigators interested in long-term outcomes.

## 1. Introduction

The first dedicated pediatric intensive care unit (PICU) was established in Europe in 1955, and pediatric intensive care has only been established as a distinct specialty since 1981 [1]. Since then, advances in the care of critically ill children have resulted in significant improvements in mortality [2,3,4,5,6]. As the vast majority of children now survive critical illness, the focus has turned to newly acquired morbidities that affect their functional abilities, health-related quality of life, and post-discharge survival [7,8,9,10,11,12,13,14]. Subsequently, our definition of a good outcome in pediatric critical care has evolved from survival, to measures of survivorship [15,16].

**Figure 1:**
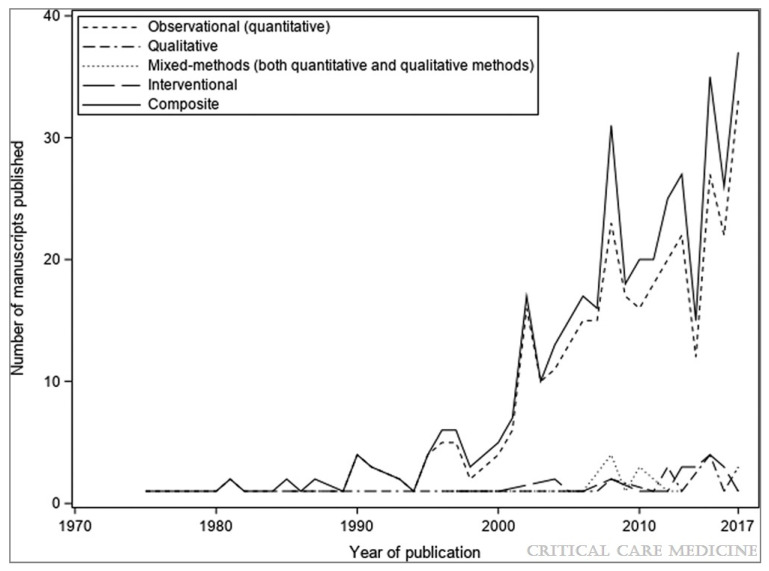
Number of manuscripts evaluating post-discharge outcomes after pediatric critical illness and study designs. *Composite* in the legend refers to the additive total number of articles. Original figure by Maddux et al. [20]. Graph generated using a comprehensive search strategy outlined in Table S2 of the Supplemental Digital Content in the original article, title and abstract exclusion, and dual full-text screening of the remaining articles [20]. Reprinted with appropriate permission from Wolters Kluwer. Original Copyright © 2021 by the Society of Critical Care Medicine and the World Federation of Pediatric Intensive and Critical Care Societies.

The evolving PICU landscape of rising morbidity and falling mortality has coincided with, and perhaps contributed to, an increasing proportion of critically ill children with underlying chronic conditions, and those with complex conditions who require prolonged PICU admissions [17,18,19]. Pediatric critical care research over the last 20 years has increasingly focused on understanding this evolving, diverse population and their outcomes beyond the PICU (Figure 1) [20]. This research has revealed that PICU survivors experience a constellation of post-intensive care morbidities that can significantly impact their long-term outcomes. In this narrative review, we describe the epidemiology of the post-intensive care syndrome in pediatrics (PICs-P), risk factors, and management strategies, how PICs affect caregivers and families, and long-term outcomes. We identify related research priorities including ongoing efforts to derive an internationally relevant core-outcome set for clinical research in the field of pediatric critical care.

## 2. Post-Intensive Care Syndrome in Pediatrics

In 2010, stakeholder meetings were held in North America with representatives from key professional organizations and groups, aiming to better define and address the lasting impairments experienced by adult intensive care unit (ICU) survivors [21]. One of the initial goals of these meetings was to establish a common nomenclature describing post-ICU morbidity to facilitate future collaboration, research, and education. It was recognized that many ICU survivors experienced co-morbidities affecting physical, cognitive, and psychiatric domains. This phenomenon was coined the “post-intensive care syndrome” (PICs) to identify the presence of one or more of these ICU-acquired sequelae [21]. The effect of intensive care hospitalization on family members was also recognized. The term “PICs-Family” emerged to recognize psychiatric sequelae such as post-traumatic stress disorder in relatives and caregivers [22].

**Figure 2:**
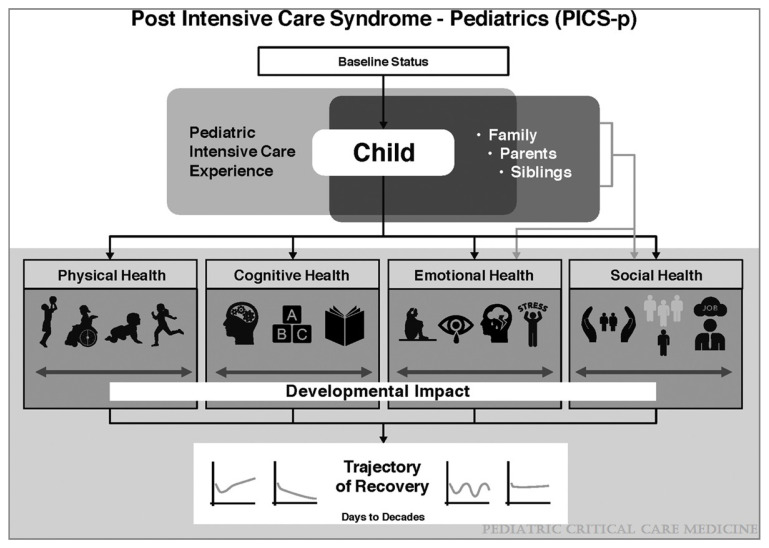
A framework for the post-intensive care syndrome—pediatrics (PICS-p). Original figure by Manning et al. [23]. Reprinted with appropriate permission from Wolters Kluwer. Original Copyright © 2021 by the Society of Critical Care Medicine and the World Federation of Pediatric Intensive and Critical Care Societies.

To address emerging evidence of post-PICU morbidity in children, Manning et al. adapted the PICs conceptual framework to pediatrics (PICs-P) (Figure 2) [23]. The pediatric model differs from that in adults in a number of ways. Residual morbidity in children following critical illness can affect four health domains: physical, cognitive, emotional, and social (examples in Table 1). These domains are influenced by the interplay of the child’s pre-morbid baseline status, the pediatric intensive care experience, and the caregiver or family unit. The social domain was added based on evidence that critical illness affects children’s post-discharge social functioning [24,25,26]. PICs may manifest differently in children due to dynamic states of growth and development, and the presence of pre-existing complex or chronic conditions. Critically ill children may therefore experience more heterogeneous trajectories of recovery than what is seen in adults [27,28].

## 3. Physical Manifestations

One of the earliest aspects of adult PICs described in the modern cannon of medicine was critical illness associated polyneuromyopathy, or “ICU-acquired weakness” [29,30,31,32]. ICU-acquired weakness is extremely challenging to ascertain in children and likely under-recognized [33]. Furthermore, weakness is not only a reflection of muscle function or power but is inextricably linked to neurologic function, pain, heart function, and lung function [28,34,35,36]. “Functional status” or “functional outcomes” are thus a more patient-centered means of conceptualizing the physical health domain than mobility or strength alone. Physical functioning assessments are now commonly measured as a part of multidimensional scales of movement, activity, endurance, abilities, and health-related quality of life [37]. This is of particular importance in a developmentally heterogeneous PICU population. Functional outcome has not only important implications for the patient, but for resource utilization [38]. Functional morbidity is associated with increased critical care resource utilization and prolonged length of hospital stay [35,39,40]. Poor functional outcomes at discharge, including technology dependence and complex chronic conditions, increase the risk of unplanned readmission and post-discharge mortality [41,42,43].

Functional sequelae following critical illness are widely dependent on the developmental stage and baseline function of the patient in question [36]. For infants or children with severe neuromuscular problems at baseline, movement may be very poor, and more salient indicators of overall function may be a decline in respiratory status ranging from subjective dyspnea or difficulty with secretion clearance to the need for positive pressure ventilation. Similarly, feeding issues might arise ranging from normal intake to mild dysphagia to dependence on parenteral nutrition. This wide variety of problems composing functional outcomes is apparent in newer tools used to measure them [44].

The prevalence of physical functional decline after critical illness is variable depending on the methods of and timing of assessment [12,34,40]. Functional status decline is reported in 4.6–10.3% of children at PICU discharge when assessed by objective, provider-completed instruments [34,35,45,46,47]. Interestingly, when disability inventories were completed by parents and caregivers at discharge, a striking 81.5% of children were identified as having had functional decline compared with their baseline prior to intensive care [40]. This finding suggests that parent perception of disability may be higher than what is uncovered with brief clinician-applied scoring tools. Functional recovery trajectories in children with acquired disability vary according to study and methods of functional assessment. Choong et al. found that approximately two-thirds of affected children demonstrated some functional recovery by 6 months post-PICU discharge [40]. In a large clinical trial population followed for a median of 4 years, 56% demonstrated functional improvement, with nearly 82% recovered to baseline function or mild dysfunction from baseline [12,36]. Conversely, some studies have shown accumulation of disability over time [47,48]. Telephone follow-up with patients consecutively admitted to a PICU demonstrated that functional morbidity continued to accumulate over a 3-year follow-up period, increasing from 5.2% to 10.4% of patients [35]. Subpopulations can have much higher rates of functional decline. In sepsis and traumatic brain injury survivors, 34% and 51% had functional decline, respectively [49,50]. However, both groups showed favorable recovery in 47% and 95% of patients, respectively, after 1 year [51,52]. Despite longer median lengths of stay in the PICU and more preceding medical complexity, children with preceding feeding or respiratory technology dependence have similar likelihood of functional decline to their less medically complex peers, 4.5 vs. 4.6% [53].

## 4. Cognitive Manifestations

Cognitive manifestations following critical illness include deficits in attention, memory, or processing speed. The true prevalence of cognitive decline following critical illness in children is challenging, as baseline neurocognitive testing is often not available nor feasible [20]. Assessments are often by basic objective scoring, caregiver recall, teacher survey, or the use of age-matched controls [54,55,56,57], and hence, measurement of cognitive decline may be prone to recall bias [46,58]. Non-verbal children and those with neurocognitive disabilities present additional challenges and are under-represented in the existing literature [59].

Based on United States database data, the overall rate of newly acquired cognitive disability following critical illness is 3.4% [46]. A recent systematic review covering cognitive outcomes in critically ill children reports considerable heterogeneity [59]. Subpopulations at highest risk are children with sepsis and meningitis, with 22.5–42% reported as having cognitive or neurodevelopmental delays following their illness [59]; among children with post-cardiac extracorporeal membrane oxygenation (ECMO), 46.8% are reported as having learning difficulties and 29.2% requiring some form of special education [55], and 24% of young children surviving extracorporeal cardiopulmonary resuscitation (E-CPR) have intellectual disability [60].

Longer-term follow-up of cognitive outcomes in children following PICU admission has demonstrated concerning findings that these deficits may persist. In children demonstrating neurocognitive impairment at 3–6 months, deficits were found to persist at 12-month follow-up [61]. Neurocognitive follow-up in pediatric survivors of meningococcal sepsis treated from 1988 to 2001 at a median 13 years following index intensive care admission demonstrated that measured intelligence quotient was comparable to the general population but had lower scores on verbal and numerical fluency subtests with effect sizes of 10–20% [57]. The effects of these cognitive changes on school performance, educational attainment, and health-related quality of life are unknown, and additional studies are needed. The importance of even minor cognitive decline should not be overlooked as it is thought to be one of the most salient factors determining health-related quality of life in PICU survivors [62].

## 5. Emotional and Psychological Manifestations

Emotional sequelae of PICs-P can manifest as mood, psychological, or psychiatric disorders acquired during ICU hospitalization that persist following discharge [24]. The majority of existing literature on emotional health has focused on school-aged children due to available diagnostic and reporting tools [63]. Therefore, a significant proportion of emotional morbidity might be missed in younger children. Specific mental health diagnoses, such as post-traumatic stress disorder (PTSD), depression [64,65], changes in self-esteem, delusional memories or fears, and sleep disturbances, have been reported in children following PICU discharge [64,65,66,67,68,69].

PTSD is particularly common amongst critically ill children [70]. As many as 84.6% of PICU survivors compared to 6.2% of ward survivors met criteria for probable PTSD or actual Diagnosis and Statistics Manual of Mental Disorders 5th edition (DSM5) criteria, according to an Egyptian study [71], while other studies report that 13–32% of children screen positive for PTSD within a year of discharge [27,72]. PTSD symptoms are thought to improve over time [72]. However, Colville et al. observed higher trauma scale scores at 12 months compared to 3 months in 40% of children. This suggests that for some children, peak symptom severity may not be captured with short follow-up periods [27].

Additional post-PICU psychiatric morbidities include hyperactivity or conduct issues, which have been reported to affect up to 20% [61]. Depressive symptoms are also common, with 83.1% of children self-identified as having symptoms of depression [71]. Sleep disturbances have been reported in up to 80% of PICU survivors, and 38% are at risk for fatigue disorder [61]. Cognitive fatigue, a term differentiating poor sustained attention and motivation, is significantly higher in PICU survivors compared to age-matched controls [73].

The impact of emotional decline following PICU is not yet fully understood. Nevertheless, early recognition and referral of PICU patients who are high-risk for adverse emotional outcomes, particularly those with high levels of medical traumatic stress, may improve outcomes including health-related quality of life [15,25,74,75,76]. The downstream effects of adverse emotional outcomes on trajectory of PICs-P recovery have not been well elucidated, but there is evidence to suggest that more children with psychiatric morbidity after discharge are re-admitted with physical complaints in the following 6-12 months [70].

## 6. Social Manifestations and PICs-Family

As their main social support, PICs affect not just the patient, but also the family. In turn, family wellbeing and functioning can significantly impact child outcomes. Issues ranging from social isolation of children to parental stress and loss of employment are all critical outcomes that may be predictive of the child’s recovery [24]. Qualitative studies in PICU survivors have shown themes related to living disrupted lives, experiencing social stigma, and having to rebuild social identities, particularly in older children [26,66]. Similarly, recent qualitative studies of parents of children who experienced critical illness identified themes related to prolonged adjustment periods following intensive care where they get used to a “new normal” [77,78,79]. The breadth of issues is reflected in the diversity and depth of literature on this topic. A recent scoping review on post-discharge outcomes in children found more studies related to social outcomes than any other PICs domain [20].

School attendance is an important objective measure of the impact of pediatric critical illness on a child’s social health [80]. Kastner et al. noted that among children admitted to an urban PICU, 43% of children followed up at 3 months had missed 7 or more days of school and 14% had missed more than 30 days of school [81]. The implementation of educational supports was also inadequate, with over half of absentee children not receiving homebound services and over a quarter having falling grades post-PICU. Chronic critical illness likely only exacerbates school absenteeism and social isolation [82,83].

Parental psychological health is closely linked to their child’s psychosocial wellbeing and their ability to support their child’s recovery [72,75]. In total, 21% of parents experience moderate to severe anxiety, and 9% reported moderate–severe depression [84]. Parent medical traumatic stress outcomes following pediatric critical illness are not uncommon [42], occurring in the range of 10.5% to 21% [85]. A study in the United States revealed that 9.5% received a new mental health diagnosis in the 6 months following their child’s PICU hospitalization, a 110% increase from the baseline rate of mental health diagnoses in this group prior their child’s PICU admission [86].

Studies assessing children’s peer relationships are notably lacking in the literature. Similarly, social determinates of health such as parental age, employment status, educational attainment, food and housing security, and support networks are known as predictors of parental resilience but have not been studied themselves as outcomes [84,87,88].

## 7. Patient Centered Outcomes in PICs-P

Outcomes research in the PICU has historically focused on clinician-important measures, tending to focus on short-term physiologic or laboratory markers [89]. In recent years, PICU research has increasingly focused on patient-centered outcomes, which require longer follow-up and prioritize measures that incorporate patient or family perceptions of wellness [8,90,91].

Health-related quality of life (HRQoL) is a uniquely patient-centered outcome and does not fall easily into any one PICs domains but rather spans them all. High-quality measures of HRQoL in children contain subscores for the physical, emotional, or social wellbeing of a child. Certain measures have also been validated for children with chronic complex disease with additional normative data and may therefore be highly useful in measuring outcomes across diverse PICU populations [90].

In consecutively admitted PICU patients tested following discharge, global scores on HRQoL measures have been very low [75,92]. Key determinates of poor HRQoL in children post-PICU include diagnoses such as sepsis, meningitis, trauma, and antecedent chronic comorbidities [49,54,93,94,95]. Treatments including prolonged cardiopulmonary resuscitation, prolonged length of stay, and use of invasive technology may also decrease post-discharge HRQoL [15,96,97]. However, longer-term follow-up has been more reassuring, showing that HRQoL generally improves after discharge for up to a year and in many children may recover to a point where children with histories of critical illness are not easily discernable from their healthy peers [25].

However, to date, there remains little uniformity in the specific measures used to assess PICU outcomes. A recent scoping review of the literature from 1970 to 2017 describes an exceptionally high number of instruments available for testing [20]. The unique article to unique measurement ratio reported in this review was 1.11, suggesting that, taken on average, for nearly every publication on this area, a new instrument was used. Consensus on ideal measures in each domain does not exist, but efforts to do so are underway. Fink et al. have initiated an effort to provide a core outcomes set which will direct clinicians and researchers to a smaller number of ideal tools that will screen for risk factors and measure outcomes which are important to patients and clinicians [98,99]. These efforts will improve the ability to measure outcomes of survivorship in a more cohesive manner.

## 8. Screening and Identification of At-Risk Patients

Evidence on potential risk factors for adverse long-term outcomes and PICs-P has only recently begun to emerge. Numerous interacting and collinear illness severity variables appear predictive of poor outcomes in all domains [56]. Severity of illness has been demonstrated in multiple studies to mediate the risk of all PICs domains [40,46,49]. Similarly, studies have recently described the use of objective organ dysfunction scoring to accurately predict trichotomous outcomes of mortality, survival with morbidity, and survival without new morbidity [100,101]. Perhaps counterintuitively, children with preceding chronic complex comorbid disease may have similar decline in function to previously healthy children or even be more likely to return to their pre-ICU baseline [40,53,102]. However, these children have high rates of PICU readmission, and the cumulative impact of recurrent or chronic critical illness is not well described [43]. Intensive care providers should therefore consider additional testing of patients with high severity of disease and chronic complex comorbid disease.

Screening patients for PICU-related iatrogenic and environmental risk factors is also important, as evidence suggests that these are independent predictors of poor post-PICU outcomes [40,103]. Excessive sedation, inadequate pain control, delirium, and immobility are classic risk factors associated with poor outcomes in adult ICU patients [104]. However, these factors can be difficult to disentangle from disease-related risks, since the intensity and duration of these therapies is related to disease severity and need for mechanical support [105]. Duration of sedation, use of certain opioid medications, and presence of withdrawal symptoms may be associated with worse functional and neuropsychiatric outcomes [47,106]. Despite strong evidence in the adult literature showing delirium as a risk factor for cognitive decline, the pediatric literature has yet to show a strong association of delirium with cognitive decline [107,108]. However, there appears to be a weak association of delirium with functional decline [47,103]. A prospective randomized trial of early vs. late parenteral nutrition (PN) in the PICU demonstrated that the use of PN in the first week of admission may contribute to limited, adverse neurocognitive outcomes at 2 years follow-up and adverse emotional and behavioral outcomes at 4 years follow-up [109,110]. Other treatments, such as glycemic control, do not appear to modify risk [111].

Demographic and social factors are also thought to mediate risk. A recent modified Delphi consensus of PICU and pediatric palliative care providers identified a number of candidate risk factors for adverse psychosocial outcomes following pediatric critical illness. The panel identified demographic factors including young caregivers and caregiver language barriers as highly predictive of poor outcome. Consensus was also reached regarding the importance of a variety of social determinates including the presence of social supports, involvement of child protective services, transportation challenges, and caregiver intellectual disability. Clinical factors considered most important were new technology or tracheostomy dependence and recurrent hospitalizations over the past year [88]. This may also negatively impact caregivers who are taking on a new, higher level of caregiver burden [112].

Our understanding of the epidemiology and risk factors of PICs-P remains nascent. Improved screening tests are needed to help to identify children at highest risk. Long-term follow-up of patients by PICU teams is rare, and the use of multidisciplinary follow-up programs, even more so [113]. Until better screening tools exist to reliably identify high risk patients, providers should have a low threshold for referral to available outpatient therapy and psychology.

## 9. Management and Prevention of PICs-P

Prioritization of reversal of organ dysfunction and survival is of foundational importance in critical care but cannot be viewed in isolation if one is to take a holistic view of patient outcomes [114]. Recognizing the potential role of iatrogenic harm in contributing to post-intensive care morbidities across PICs domains, care bundles have been proposed to mitigate the risks that accompany life-saving treatments. The most widely accepted in recent years has been the ABCDEF bundle [115]. 

The ABCDEF bundle is a harm-reduction tool initially used in adults to promote ICU liberation and limit chronic morbidity ([116]. This bundle of interventions includes assessing, preventing, and managing pain, both spontaneous breathing and awakening trials, choice of analgesia and sedation, assessing preventing and managing delirium, early mobility and exercise, and family engagement and empowerment. In adults, implementation of this bundle has reduced early mortality, duration of mechanical ventilation, duration of coma, incidence of delirium, physical restraint use, ICU readmission, and has increased the percentage of patients that return to their home versus chronic treatment facilities [116]. In pediatrics, evidence for this bundle is accumulating, and several recent and ongoing studies evaluating early mobilization, delirium prevention, and sedation protocols reveal promising results [117,118]. This and other research evidence has prompted emerging practice recommendations for the routine assessment of pain, sedation, withdrawal, and delirium in the PICU [119,120].

Other promising interventions that have shown benefits in adults and are planned for study in children include the use of “PICU Diaries” [121]. This intervention involves partnering with families to provide a lay narrative, comprising daily entries, drawings, and/or photographs about their child’s condition and care while in the ICU [122]. Diaries fill in memory gaps, provide a means for coherent recall of events, and may help children to make meaning out of their experience. In adults, ICU diaries have been shown to improve HRQoL and decrease PTSD, anxiety, and depression following critical illness recovery [123]. New data show the feasibility and acceptance of this intervention among families of critically ill children, and it is ripe for further study [121,122].

Multidisciplinary follow-up clinics have been proposed as a way of testing for PICU-acquired morbidities and providing comprehensive support and intervention to high-risk patients. Ongoing support after PICU and hospital discharge has been shown to be an important protective factor in prevention of psychiatric morbidity of parents and children [76]. Parents of critically ill children have indicated support for such clinics; however, when given the opportunity, only 37% choose to attend [124]. Early efforts to provide outpatient follow-up after pediatric intensive care were collaborations between pediatric neurology and pediatric intensivists caring for neurocritical care survivors [125]. These clinics have expanded to include neuropsychiatric professionals and allied health professional support [124,126]. Currently, a minority of PICUs have these types of clinics [127]. Inadequate data exist to recommend specific timing, frequency, or specialty mix of these clinics, and the limited data available have yet to show effectiveness as an intervention [124]. Additional research is needed to understand whether such care provides any significant protection or improved recovery for PICU-acquired co-morbidity.

## 10. Research Priorities

The need to better understand patient-centered outcomes and PICs-P has led researchers to prioritize how to best measure outcomes in critically ill children. PICU core outcome set (COS) investigators, in conjunction with the Pediatric Acute Lung Injury And Sepsis Investigators (PALISI) Network and the Collaborative Pediatric Critical Care Network (CPCCRN), are currently working with clinical and family stakeholders to determine ideal outcomes and associated measurement tools to evaluate PICU and long-term outcomes of children and their families [98,99]. The resulting core outcomes set and measures will serve as a guideline and resource for clinicians and potentially family caregivers and patients to track their own recovery. Researchers can also draw on ideal outcome tools to assess the efficacy of interventions targeted at optimizing PICU and long-term functional and HRQoL outcomes.

Ongoing research targeted at early rehabilitation and reducing PICU-acquired morbidities will provide increasing evidence on the efficacy of these interventions on clinical, patient-centered, as well as process of care outcomes, and how best to implement these multi-prong bundles in the PICU setting [128,129,130].

Discovery of means for improved patient retention including the use of multidisciplinary ICU follow-up clinics and other forms of post-discharge family support are critical to improving the retention of patients and validity of findings in outcomes research. As in any field, data sharing and outcomes registries will be an important component of retrospective analysis. Additionally, the continued funding of studies with aims related to long-term outcomes in large PICU trials will add to our detailed understanding of specific interventions and populations.

## 11. Conclusions

The post-intensive care syndrome in pediatrics is marked by poor outcomes across multiple domains of health, functioning and HRQoL. Pediatric intensivist, acute care clinicians, outpatient physicians, and allied health professionals should be increasingly aware of critical illness sequelae and how they affect both patient and family. Clinicians need to be able to identify patients at risk early in their critical illness course and minimize the risk of PICU-acquired complications and institute early rehabilitation. Ward clinicians, subspecialists, rehabilitation specialists, and outpatient primary care physicians should be educated on how best to screen for PICs-p symptoms and ensure adequate ongoing rehabilitation and follow-up to optimize long-term outcomes and recovery. 

The current legacy of pediatric critical care is one of great historical success in improving survival. The future of pediatric critical care is one of great promise of improving survivorship. Pediatric intensivists are looking toward a future where an increased understanding of outcomes epidemiology, risk factors, and interventions leads to reduction in PICs-P frequency and severity.

## Figures and Tables

**Table 1:** PICs-P domains and selected examples of adverse outcomes in each domain. Listed examples are not comprehensive, and some examples can span multiple domains.

PICs-P Domains	Examples		
Physical Health	Chronic organ dysfunction or failure Technology dependence Chronic pain Feeding problems or malnutrition Fatigue or weakness Sleep disturbances.	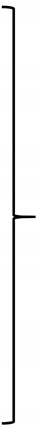	Health-Related Quality of Life
Cognitive Health	Reduced attention Memory problems Decreased communication abilities Decreased school achievement.		
Emotional Health	Depression Post-traumatic stress symptoms and disorder Anxiety Delusional memories and fears Behavioral problems Sleep disturbances.		
Social Health	Loss of peer relationships Loss of social identity School absenteeism Decreased participation Strained family relationships Social anxiety.		
PICs-F (Family)	Parent, caretaker or sibling psychiatric complications Job loss Food or housing insecurity Strained family relationships Family financial strain		

## Abbreviations/Acronyms

ABCDEF	Bundle of Care to improve ICU outcomes further defined in text
COS	Core Outcome Set
CPCCRN	Collaborative Pediatric Critical Care Research Network
DSM5	Diagnostics and Statistics Manual 5th Edition
ECMO	Extracorporeal Membrane Oxygenation
E-CPR	Extracorporeal Cardiopulmonary Resuscitation
HRQoL	Health-Related Quality of Life
ICU	Intensive Care Unit
PALISI	Pediatric Acute Lung Injury and Sepsis Investigators Network
PICs	Post-intensive Care Syndrome
PICs-P	Post-intensive Care Syndrome in Pediatrics
PICU	Pediatric Intensive Care Unit
PTSD	Post-traumatic Stress Disorder
PN	Parenteral Nutrition
